# Magnitude of Episiotomy and Associated Factors among Mothers Who Give Birth in Arba Minch General Hospital, Southern Ethiopia: Observation-Based Cross-Sectional Study

**DOI:** 10.1155/2020/8395142

**Published:** 2020-09-01

**Authors:** Kassahun Fikadu, Negussie Boti, Birtukan Tadesse, Dureti Mesele, Emenet Aschenaki, Etenesh Toka, Fistum Arega, Tsehaynesh Girma, Abebech Paulos

**Affiliations:** ^1^Midwifery Department, College of Medicine and Health Sciences, Arba Minch University, Arba Minch, Ethiopia; ^2^School of Public Health, College of Medicine and Health Sciences, Arba Minch University, Arba Minch, Ethiopia

## Abstract

**Background:**

Episiotomy is the most common obstetric procedure, performed when the clinical circumstances place the patient at a high risk of high-degree laceration. However, episiotomy should be done with judicious indication to lower perineal laceration with fewer complications. Despite its adverse effects, the magnitude of episiotomy is increasing due to different factors. Therefore, this study is aimed at determining the recent magnitude of episiotomy and at identifying associated factors among women who gave delivery in Arba Minch General Hospital, Southern Ethiopia.

**Methods:**

An institution-based cross-sectional study was conducted from December 15, 2018, to January 30, 2019. A systematic random sampling technique was used to select study participants. A semistructured questionnaire was used to collect data. This was supplemented with a review of the labor and delivery records. Binary and multivariable logistic regression analyses were performed to identify factors associated with the magnitude of episiotomy. *P* value ≤ 0.05 was used to determine the level of statistically significant variables.

**Results:**

The magnitude of episiotomy was found to be 272 (68.0%) with 95%CI = 64.0‐72.5. Women who attended secondary education [AOR = 10.24, 95%CI = 2.81‐37.34], women who attended college and above [AOR = 4.61, 95%CI = 1.27‐16.71], birth weight ≥ 3000 g [AOR = 4.84, 95%CI = 2.66‐8.82], primipara [AOR = 4.13, 95%CI = 2.40‐7.12], being housewife occupants [AOR = 3.43, 95%CI = 1.20‐9.98], married women [AOR = 2.86, 95%CI = 1.40‐5.84], and body mass index < 25 kg/m^2^ [AOR = 2.85, 95%CI = 1.50‐5.44] were independent variables found to have significant association with episiotomy.

**Conclusion:**

The magnitude of episiotomy was 68.0% which is higher than the recommended practice by WHO (10%). The study participants' occupational status, marital status, educational status, parity, birth weight, and BMI were significantly associated with the magnitude of episiotomy in the study area. Therefore, to reduce the rate of episiotomy, it is better to have periodic training for birth attendants regarding the indication of episiotomy.

## 1. Background

An episiotomy is one of the widely used obstetric interventions which is done by the birth attendant to minimize the risk of severe tears which occur due to enlarging of the birth canal during childbirth at a time when the fetus's head descends [1, 2].

The American College of Obstetricians and Gynecologists (ACOG) and Federation of International Gynecology and Obstetricians recommend that episiotomy should be done with judicious indication to lower perineal laceration with fewer complications [3, 4]. Existing evidence also supported the recommendation to restrict episiotomy use [5].

The finding from studies conducted in a different part of the world shows that episiotomy increases the risk of third- and fourth-degree perineal lacerations which had short- and long-term complications for mothers [6–8]. A study conducted in Taiwan indicated that episiotomy increased the number of women who had pain at the first, second, and sixth weeks of postpartum and urinary incontinence [9]. Most of the consequences of episiotomy affect the parturient, greatly impacting her quality of life and leaving her with an unpleasant childbirth memory [10]. Besides, the findings show that the perineal tear and pelvic floor morbidity can be increased among women receiving episiotomy [11, 12]. Episiotomy use is associated with a higher incidence of perineal pain in the immediate postpartum period, where it predisposes them to risk of psychological morbidity and stress urinary incontinence in 6 weeks postpartum.

Despite its adverse effects, the magnitude of the episiotomy is increasing due to different factors [13]. Findings from studies conducted in India and China show that the magnitude of episiotomy continues to be high which range from 60% to 80% [13–15]. It also continues to be high in developing countries [1, 16, 17].

The finding from studies conducted in different parts of Ethiopia revealed that the magnitude of episiotomy became over 30% and the practice was reported to increase up to 2.3-folds more in a rural part of Ethiopia [15, 17]. The rate of episiotomy practice reported was significantly higher than recommended, noting that perineal repair without analgesia needs to be revised and a less painful method should be advocated [18]. Moreover, a national health facility report in Ethiopia indicated that episiotomy alone had caused 9% and 8% of primary postpartum hemorrhage and maternal sepsis, respectively [19].

The magnitude of episiotomy practice varies according to the obstetric procedure, maternal and fetal conditions, type of birth attendant, level of education, and years of experience of birth attendant. Therefore, this study is aimed at determining the recent magnitude of episiotomy and at identifying associated factors among women who gave delivery in Arba Minch General Hospital, Gamo Zone, southern Ethiopia, which may help to reduce adverse consequences to the mother. Moreover, the finding of this study may help clinicians to make an informed decision about episiotomy-related clinical practice, thereby achieving the best pregnancy outcome.

## 2. Methods

### 2.1. Study Area, Period, and Design

An institution-based, cross-sectional study was conducted from December 15, 2018, to January 30, 2019, in Arba Minch General Hospital. Arba Minch is an administrative town of the Gamo Zone, located about approximately 500 km south of Addis Ababa, the country's capital city. It consists of 11 Kebele (the smallest administrative unit) with a total population of 112,724. There are 26,265 reproductive age group [14] women residing in the town of whom 4428 were pregnant and 3261 were giving birth at the facility level. There is one general hospital, two health centers, and 17 primary and 14 medium private clinics in the town. Arba Minch Hospital is teaching center in different disciplines and specialty areas for medicine and health science students ..

### 2.2. Source and Study Population

Those mothers who gave birth in Arba Minch General Hospital during the specified study period were eligible for the study. However, mothers who underwent destructive delivery were excluded from this study.

### 2.3. Sample Size Determination and Sampling Procedure

The sample size was estimated using a single population proportion formula considering the following assumptions: the magnitude of episiotomy (*P* = 41.4%) from the study conducted in public health institutions of Axum Town, Northern Ethiopia [20]; confidence level of 95%; 5% of the margin of error; and 10% nonresponse rate. As a result, the calculated sample size for this study was 410. To select study participants, the systematic random sampling technique was employed. The sampling process was stopped when the required sample size was met. The sampling interval was determined based on the monthly average number of deliveries. Accordingly, the hospital report in the year 2018 of the average monthly number of deliveries was 1315 (i.e., *K*^th^ value 1315/410 = 3.2 ~ 3). The initial mother was picked by lottery method, then the next mother was selected every three intervals according to their order of admission to labor until the final sample size was fulfilled.

### 2.4. Data Collection Method

Data was collected using a pretested, interviewer-administered, semistructured questionnaire. This tool was developed from similar studies conducted in a different part of the world [17, 20–27]. The questionnaire was primarily developed in English, translated into Amharic (local language), then translated back to English and rechecked by the third person, to ensure its correctness and consistency. The interview questionnaire consisted of four key items: sociodemographic characteristics of participants, labor- and delivery-related factors, and maternal- and fetal-related variables. Fetal gestation and weight were collected from the maternal follow-up sheet.

Seven BSc midwives who had experience in data collection were selected for data collection and three MSC midwifery students supervised the data collection. All data collectors were responsible for observing respondents starting from the commencement of the active first stage of labor to the occurrence of the outcome. Then, the secondary data extraction follows until the patient has had a stable vital sign and her medical condition has been confirmed for interview by a duty physician. Then, the interview was conducted in a place where the mother's privacy and comfort could be kept. All interviews were conducted in the local language. The data collectors were supervised by MSC midwifery students based on hospital ward rotation. The data collectors were scheduled in every shift to collect data from the admission of parturient to the end of the first two hours after childbirth through an interview, observation, and delivery records. The data collectors used a unique numeric identifier to track the mothers' card back during data extraction. They were instructed to put this code on each questionnaire's front page. Each time during the data collection phase, between 5:00 and 5: 30 pm, we had a regular meeting to discuss challenges. Then, the research was notified right away to make a solution for the next day. The interviews ranged from 10 to 15 min per participant. All study subjects were permitted to be interviewed.

### 2.5. Data Quality Management

To control data quality, the questionnaire was pretested in 5% of the sample size among women who gave birth in Gidole Hospital. A minor amendment on consistency, coherence, and skipping patterns was made after a pretest was conducted. Besides, both the data collectors and supervisor had been given one-day training on how to complete questionnaires, interview puerperal patients, and extract data from the delivery registration by the researcher. During the data collection phase, the supervisor checked the completeness of the questionnaire each day.

### 2.6. Data Analysis

The collected data were coded, entered, and cleaned by using Epi Info version 7.2.0 software. Then, it was exported to SPSS version 20. Descriptive statistics were carried out and summarized by tables, frequencies, graphs, and means. An association between the magnitude of episiotomy and potential factors was examined using binary logistic regression. The odds ratio and confidence interval were calculated to determine the strength of the association. From the bivariable analysis, those variables *P* ≤ 0.25 and biological plausibility were potential candidates for multivariable logistic regression analysis. We check multicollinearity between variables and outcome variables by using a variance inflation factor (VIF). Variables with a VIF greater than 10 were dropped from the candidate variables to be fitted into the final model. The goodness-of-fit was assessed using the Hosmer-Lemeshow test. Variables with a nonsignificant Pearson chi-square test but a significant omnibus test were considered eligible to be fitted to the multivariable model. Variables with a *P* value ≤ 0.05 in the multivariable logistic regression model were considered statistically significant. Finally, the significance of an association between episiotomy received and independent variables was reported with corresponding 95% CI.

## 3. Results

### 3.1. Sociodemographic Characteristics of the Study Subjects

Out of 410 mothers who were expected to participate, 400 mothers participated in this study, which gave a 97.6% response rate. The majority of 193 (48.3%) of the study subjects were less than 27 years of age with a 3.9 standard deviation. Of all study subjects, more than 85% were married. Most of the 255 (63.8%) study subjects in this study lived in an urban area. More than 29% of the study subjects were either government or self-employees. About 26.8% of the study participants had completed at least a college education (Table 1).

### 3.2. Labor- and Delivery-Related Characteristics

In this study, about 207 (51.8%) respondents had given birth during the night time. More than 39% of the respondents had given birth assisted by a vacuum extractor. Regarding duration of labor, 224 (56%) laboring mothers stayed more than 7 hours in the first stage of labor. However, 222 (55.5%) of laboring mothers took less than 2 hours to deliver their neonates after commencement of the second stage. Two hundred and thirty-four, 58.5%, of study subjects had given birth assisted by midwife professionals (Table 2).

### 3.3. Maternal-Related Characteristics

Of the total multiparous women in this study, 91 (48.4%) of them had a history of previous episiotomy being performed. Among mothers who had pregnancy above 28 completed weeks, 60 (31.9%) of them had a history of previous breech delivery. Among the total respondents, the majority, 325 (81.3%) respondents, had female genital mutilation. About 212 (56%) of the respondents had given birth for the first time. Approximately fifty-two percent of the respondents were found to have a body mass index of more than the optimal range. In 103 (25.8%) mothers, a previous history of chronic illness was reported (Table 3).

### 3.4. Fetal-Related Characteristics

Out of the total delivery, 298 (74.5%) were born at the beginning of 37 and above completed weeks of gestation. The majority of 363 (90.8%) of the reported fetal presentation was cephalic. During the current study, the fetal condition was described by the presence of clear amniotic fluid in 308 (77%) of the respondents. More than half of the delivered neonates had less than 3300 gm (IQR ± 2000) (Table 4).

### 3.5. Magnitude of Episiotomy

**Figure 1:**
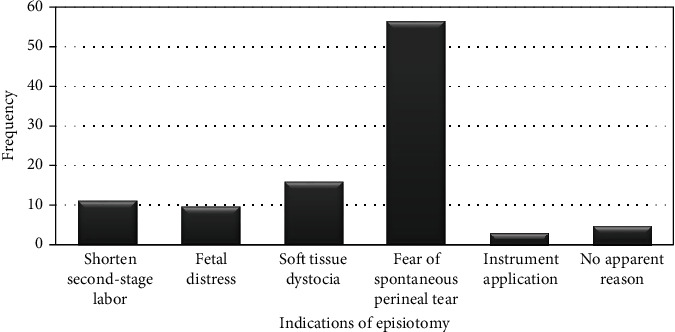
Indication of episiotomy among mothers who gave birth in Arba Minch General Hospital, 2019.

The findings of this study revealed that the magnitude of episiotomy was found to be 272 (68.0%) with 95%CI = 64.0‐72.5. In this study, the main reason for performing episiotomy procedure was fear of spontaneous perinatal laceration, accounting for 55.8%, followed by 15.8% where it was rated for soft tissue dystocia (Figure 1).

### 3.6. Factors Associated with the Magnitude of Episiotomy

From the bivariable analysis, those variables *P* ≤ 0.25 and biological plausibility were potential candidates for multivariable logistic regression analysis. Variables like marital status, maternal age, parity, educational status, occupation, birth attendant, duration of second-stage labor, residence, gestational age, time of delivery, history of FGM, estimated fetal weight, history of chronic illness, and body mass index were fitted in the final multivariable model. Those variables like occupation, BMI, birth weight, parity, marital status, and educational status were significantly associated with the outcome variable in the final multivariable analysis.

In this study, those women who were housewife occupants were found to have a significant statistical association, where being a housewife occupant was more than 3.4 times more likely to get incised during delivery than student mothers [AOR = 3.4, 95%CI = 1.2‐9.9]. Mothers who had married were 2.9 times more likely to be incised during delivery than those mothers who were unmarried [AOR = 2.9, 95%CI = 1.4‐5.9]. Mothers' educational status was another independent variable which found to have a statistically significant association with increased magnitude of episiotomy performance. Mothers who attended secondary education [AOR = 10.2, 95%CI = 2.8‐37.3] and those mothers who attended college and above [AOR = 4.6, 95%CI = 1.3‐16.7] were compared to those mothers who did not attend formal education.

On the other hand, mothers who were primiparous were four times more likely to incur episiotomy procedures than those who were multiparous [AOR = 4.1, 95%CI = 2.4‐7.1]. Mothers who gave birth to a neonate whose weight was more than or equal to 3300 gm were 4.8 times more likely to incur an episiotomy procedure during delivery than those of newborns whose weight was below 3300 gm [AOR = 4.8, 95%CI = 2.7‐8.8]. Another explanatory variable that had a significant association with the magnitude of episiotomy in this study was maternal body mass index (BMI). Those mothers whose BMI was <25 kg/m^2^ were nearly three times more likely to incur an episiotomy procedure during delivery than those mothers whose BMI was ≥25 kg/m^2^ [AOR = 2.9, 95%CI = 1.5‐5.4) (Table 5).

## 4. Discussion

The primary objectives of this study were to determine the magnitude of episiotomy and to identify associated factors among women who give birth in Arba Minch General Hospital. As a result, while more than half of women who give birth incurred episiotomy during delivery time, the study participants' occupational status, marital status, educational status, parity, birth weight, and BMI were significantly associated with the magnitude of episiotomy in the study area.

The magnitude of episiotomy was 68.0% (95%CI = 64.0‐72.5). This finding is lower than the study conducted in Uganda (73%) [28] and in northern Nigeria (89.3%) [29]. However, this finding is higher than the study conducted in Vietnamese-born women in Australia (29.9%) [27]; Nigeria (21%) [30]; Brazil 29.1% [22]; Iran 41.5% [31]; Kano, Nigeria 41.4% [32]; Nepal 22% [33]; East African women in Australia 30% [34]; Eastern Nigeria 45% [23]; Mizan Aman 30.6% [17]; Addis Ababa 40.2% [16]; Shire 41.4% [20]; and Jimma 25% [15]. This difference might be due to the difference in time of the studies conducted, study settings of the study participants, and characteristics of the study population. The high prevalence may be due to the characteristics of the study participant since Arba Minch Hospital was a referral center for three catchment zones. Most women who attend labor in this hospital were high risk and most often referred to with complications; this may increase the risk of episiotomy to shorten the second stage of labor. Another high magnitude of episiotomy might be associated with experiences of birth attendants, which may suggest a more restrictive use at the study center. Also for birth attendants to reduce the episiotomy rate, applicable perineal massage, use of certain birthing positions (e.g., hands and knees), and labor support [35] are suggested. Furthermore, the high magnitude may be associated with operative delivery. In our study, we did not exclude those mothers whose delivery was by vacuum and forced. Evidence shows that operative delivery will increase the rate of episiotomy [36]. Besides, the finding of this study gives a hint on the need for training for professionals in the practice of episiotomy to lower the magnitude of episiotomy.

The finding of this study also revealed that those women whose occupational status was housewives and those women who were married were more likely incised during delivery. This finding is supported by evidence from a study conducted in Mizan Aman Hospital [17].

The educational status of women was also one of the factors with statistically significant associations with an increased magnitude of episiotomy performance. Those women who had attended secondary education and those mothers who attend college and above were more likely incised during delivery compared to those mothers who did not attend formal education. This finding was supported by a study conducted in Iran [31]. This may be due to being educated allows having information or getting aware of the needed interested area. This, in turn, may influence the study subjects to develop a fear towards episiotomy that may influence obstetric caregivers performing episiotomy [37]. This finding also gives a hint for the health profession to apply the WHO recommendation without the influence of mothers.

The parity of respondents was also one of the risk factors for the episiotomy. Those women who were primiparous were more likely to have episiotomy than multiparous women. This finding was supported by studies conducted in Brazil [22], France [8], East African migrants in Australia [34], Taiwan [38], Iran [31], Vietnamese-born women in Australia [27], and Jimma [15]. This might be since most of the time, primiparous women were prone to perineum tightening which is one indication of episiotomy, and the old recommendation of routine episiotomy in primiparous women performed by many health professionals might still have an influence in the indication of this procedure for those women [20].

Birth weight of the newborn had a significant statistical association with episiotomy, where mothers whose newborns had median weight which was equal and more than 3300 mg were 4.8times more likely to have an episiotomy during parturition. This is in agreement with studies conducted in Israel [39], Nigeria [25], Austria [40], Spain [41], Thailand [42], USA [43], and Japan [44]. However, there was no association reported from a study conducted in Jimma [15] and Iran [31]. This finding gives a hint for clinicians would tend to give episiotomy for a fetus if they assumed the weight was higher. In fact, the higher the estimated fetal weight, the more it could predispose for perinatal trauma if the provider tends to give judicious episiotomy in time. Evidence indicated that one of the main reasons why clinicians used to perform episiotomy was fear of a perineal tear [37]. Training should be given for birth attendants on the indication of episiotomy; this may reduce the fear of the birth attendant. Also, those who fear perineal tear should consult early for an experienced birth attendant.

In the current study, women whose body mass index was less than 25 kg/m^2^were nearly 3 times more likely to have an episiotomy. This is supported by a study conducted in the UK [45], New Mexico [46], and USA [47]. The risk of episiotomy was lower in women who have had an increased BMI [48]. An increased BMI at enrolment was associated with a reduced incidence of minor perinatal trauma at delivery [49]. Obese women were less likely to use tobacco, were more likely to have their labor augmented or induced with oxytocin, and had shorter second stages than women who were not obese [46]. This finding gives a hint for the health professional to follow strictly to those women who were obese.

## 5. Limitation of the Study

As this study was exclusively conducted in the hospital, the findings cannot be generalized to all women who attend labor in Ethiopia. Besides, there may be social desirability bias since we collected the data by using the interviewer administer technique. The cause and effect relationships in this study could not be determined due to the use of the cross-sectional study design for this study. Thus, we strongly recommend further study by using a better study design to ascertain cause relationships.

## 6. Conclusion

In conclusion, this study found that more than half of the study participants have had an episiotomy.

Gravidity, occupational status, marital status, educational status, birth weight of the neonate, and BMI of women were significantly associated with the magnitude of episiotomy. Therefore, different stakeholders working on maternal health programs should work on those factors to reduce the magnitude of episiotomy. Furthermore, it is better to give episiotomy-restrictive interventions to birth attendants. Moreover, clinicians and any responsible body should critically follow the work done in the hospital.

## Figures and Tables

**Table 1:** Sociodemographic characteristics of respondents on magnitude and factors of episiotomy in Arba Minch General Hospital, Southern Ethiopia.

Variables	Frequency (*n*)	Percentage (%)
Age classification		
<27 years	262	65.50
≥27 years	138	34.50

Marital status		
Unmarried	57	14.20
Married	343	85.80

Resident		
Urban	255	63.80
Rural	145	36.20

Occupation		
Housewife	97	24.30
Employee	117	29.30
Merchant	104	26.00
Student	82	20.40

Educational states		
Nonformal education	97	24.30
Primary	99	24.80
Secondary	97	24.30
Collage and above	107	26.80

**Table 2:** Labor- and delivery-related characteristics of the study on magnitude and associated factors of episiotomy in Arba Minch General Hospital, Southern Ethiopia, 2019.

Variables	Frequency (*n*)	Percentage (%)
A shift of delivery completion		
Night	207	51.80
Day	193	48.20

Vacuum extractor application		
Yes	153	39.20
No	247	61.80

Duration of first-stage labor		
Less than 7 hours	176	44.00
Greater or equal to 7 hours	224	56.00

Duration of second-stage labor		
≤2 hours	222	55.50
>2 hours	178	45.50

Birth attendant		
Midwife	234	58.50
Other health care provider	166	41.50

**Table 3:** Maternal-related characteristics of the study on magnitude and associated factors of episiotomy in Arba Minch General Hospital, Southern Ethiopia, 2019.

Variables	Frequency (*n*)	Percentage (%)
Previous episiotomy (*n* = 188)		
Yes	91	48.40
No	97	51.60

Previous breech delivery (*n* = 188)		
Yes	60	31.90
No	128	68.10

Female genital mutilation		
Yes	325	81.30
No	75	18.70

Parity		
Primipara	212	53.00
Multipara	188	47.00

Body mass index		
<25 kg/m^2^	193	48.30
≥25 kg/m^2^	208	51.80

History of chronic illness		
Yes	103	25.80
No	297	74.30

**Table 4:** Fetal-related characteristics of the study on magnitude and associated factors of episiotomy in Arba Minch General Hospital, Southern Ethiopia, 2019.

Variables	Frequency (*n*)	Percentage (%)
Gestational age		
Preterm	102	25.50
Term and above	298	74.50

Fetal presentation		
Cephalic	363	90.80
Breech	37	9.20

Liquor status		
Clear	308	77.00
Bloody	60	15.00
Meconium	32	8.00

Fetal weight		
<3300 gm	175	43.80
≥3300 gm	225	56.20

**Table 5:** Bivariable and multivariable logistic regression analysis outputs of the factors associated with the magnitude of episiotomy for mothers who gave birth vaginally in Arba Minch General Hospital, 2019 (*N* = 400).

Variables	Magnitude of episiotomy		COR (95% CI)	AOR (95% CI)
	Yes	No		
Occupation status				
Housewife	81 (83.5)	16 (16.5)	1.9 (1.3–4.4)	3.4 (1.2–9.9)^∗^
Employee	75 (64.1)	42 (35.9)	0.7 (0.8–2.8)	1.5 (0.5–4.3)
Merchant	56 (53.9)	48 (46.2)	0.4 (0.3–1.1)	1.3 (0.5–3.7)
Students	60 (73.2)	22 (26.8)	1	1

Marital status				
Unmarried	26 (45.6)	31 (54.4)	1	1
Married	246 (71.7)	97 (28.9)	3.0 (1.7–5.4)	2.9 (1.4–5.8)^∗^

Educational status				
No formal education	52 (53.6)	45 (46.4)	1	1
Primary education	68 (68.7)	31 (31.3)	1.9 (1.1–3.4)	1.8 (0.8–3.9)
Secondary education	86 (88.7)	11 (11.3)	6.8 (3.2–14.2)	10.2 (2.8–37.3)^∗^
College and above	66 (61.7)	41 (38.3)	1.4 (0.8–2.4)	4.6 (1.3–16.7)^∗^

Parity				
Primiparous	171 (80.7)	41 (19.3)	3.6 (2.3–5.6)	4.1 (2.4–7.1)^∗^
Multiparous	101 (53.7)	87 (46.3)	1	1

Birth weight				
<3300 gm	83 (47.4)	92 (52.6)	1	1
≥3300 gm	189 (84.0)	36 (16.0)	5.8 (3.7–9.3)	4.8 (2.7–8.8)^∗^

Body mass index				
<25 kg/m^2^	160 (83.3)	32 (16.7)	4.3 (2.7–6.8)	2.9 (1.5–5.4)^∗^
≥25kg/m^2^	112 (53.9)	96 (46.2)	1	1

NB: ^∗^Significant at *P* value ≤ 0.05. COR: crude odds ratio; AOR: adjusted odds ratio; CI: confidence interval; 1: reference group.

## Data Availability

The [SPSS/EXCEL] data used to support the findings of this study are available from the corresponding author upon request.

## Abbreviations

ANC: Antenatal care
AOR: Adjusted odds ratio
BMI: Body mass index
COR: Crude odds ratio
EFW: Estimated fetal weight
WHO: World health organizations.
